# Short Communication: Management of patients with extensive-stage small-cell lung cancer treated with radiotherapy: A survey of practice

**DOI:** 10.1016/j.ctarc.2018.08.004

**Published:** 2018

**Authors:** Kate Haslett, Dirk De Ruysscher, Rafal Dziadziuszko, Matthias Guckenberger, Cecile Le Pechoux, Ursula Nestle, Ben Slotman, Corinne Faivre-Finn

**Affiliations:** a Manchester Cancer Research Centre, Division of Cancer Sciences, School of Medical Sciences, Faculty of Biology Medicine & Health, University of Manchester, Manchester Academic Health Science Centre, The Christie NHS Foundation Trust, Manchester, UK; b Department of Radiation Oncology (MAASTRO Clinic), Maastricht University Medical Centre, School for Oncology and Developmental Biology (GROW), The Netherlands; c KU Leuven, Radiation Oncology, Belgium; d Department of Oncology and Radiotherapy, Medical University of Gdansk, Gdansk, Poland; e Department of Radiation Oncology, University Hospital Zurich (USZ), Switzerland; f Department of Radiation Oncology, Gustave Roussy Cancer Campus, Institut d’ Oncologie Thoracique, Villejuif, France; g Klinik für Strahlentherapie und Radiologische Onkologie, Kliniken Maria Hilf, Mönchengladbach, Germany; h Department of Radiation Oncology, VU University Medical Center, Amsterdam, The Netherlands

**Keywords:** Extensive stage small-cell lung cancer, Radiotherapy, Survey, European practice

## Abstract

**Objectives:**

The results of the randomized phase 3 CREST trial evaluating the use of thoracic radiotherapy for extensive-stage small-cell lung cancer (ES-SCLC) were published in the Lancet in 2015. The primary endpoint (10% overall survival difference at 1-year) was not achieved, but there was significant improvement in 2-year overall survival (13% vs 3%; *p* = 0.004) and low toxicity rates, suggesting thoracic radiotherapy should be considered for ES-SCLC patients who respond to chemotherapy. Questions have been raised as to whether these results will lead to a change in practice.

**Materials and methods:**

We developed an electronic survey to determine the impact of the publication on clinical practice across some European countries.

**Results and conclusion:**

We report the results of our survey, which suggest the CREST trial has changed practice, resulting in an increase in the use of thoracic radiotherapy amongst the surveyed centers from 25% to 85%. Furthermore the dose and fractionation schedule used in the trial has been widely adopted across Europe.

## Highlights

• Increased use of thoracic radiotherapy in ES-SCLC following CREST trial.
• Trial schedule of 30 Gy in 10 for thoracic radiotherapy has been widely adopted.
• High consistency in the use of prophylactic cranial irradiation.

## Introduction

Most patients with small-cell lung cancer present with extensive-stage disease and have a 2-year survival of less than 5%. Standard treatment is four to six cycles of platinum-based chemotherapy, a regime unchanged over recent decades. In an EORTC trial, prophylactic cranial irradiation administered to those who have responded to chemotherapy, reduced the incidence of symptomatic brain metastases by over half and improved 1-year survival by 14% [1].

Persisting intrathoracic disease after completing chemotherapy is common, with approximately 90% of patients presenting with intrathoracic progression within a year of diagnosis [1]. Therefore the next logical step was to investigate the role of thoracic radiotherapy in this group of patients.

The results of the randomized phase 3 CREST trial evaluating the use of thoracic radiotherapy for extensive-stage small-cell lung cancer (ES-SCLC) were published in 2015 [2]. The CREST study enrolled 495 patients from 42 centres, mainly in the Netherlands and United Kingdom. The main eligibility criteria included Eastern Cooperative Oncology Group (ECOG) scale performance status 0–2, confirmed ES-SCLC defined as disease beyond the hemithorax, hilar, mediastinal and supraclavicular nodes, any response after 4–6 cycles of platinum-based chemotherapy without evidence of disease progression at any site; and no clinical evidence of brain, leptomeningeal or pleural metastases. Patients were randomly assigned (1:1) to either thoracic radiotherapy (30 Gy in 10 fractions over two weeks) plus prophylactic cranial irradiation or prophylactic cranial irradiation only. Before randomization, patients had a CT thorax and upper abdomen. Brain imaging with CT or MRI was also performed in any patient with symptoms suspicious for brain metastases. The primary endpoint (10% overall survival difference at 1-year) was not achieved, but there was significant improvement in 2-year overall survival (13% vs 3%; *p* = 0.004), suggesting thoracic radiotherapy should be considered for ES-SCLC patients who respond to chemotherapy. Furthermore severe treatment-related toxicity was uncommon with 10.5% and 7.2% of patients developing grade 3 toxicity in the thoracic radiotherapy and the control group respectively. These results have raised controversy regarding the implementation of thoracic radiotherapy [3], [4], [5], [6], [7], [8], [9], which led to the development of a survey in some European countries to determine the impact of the publication on clinical practice.

## Materials and methods

In May 2015, an electronic questionnaire (see Appendix 1) comprising 34 items was developed using publicly available software.

Questions included in the survey:

• Use of thoracic radiotherapy before and after the publication of the CREST study results in different clinical scenario (symptomatic residual disease, asymptomatic central residual disease, no central disease)
• Practice of prophylactic cranial irradiation in ES-SCLC
• Reasons for not implementing thoracic radiotherapy
• Staging investigations performed
• Future research questions

One academic lead in radiation oncology per country circulated the survey by email to all centres within their country. It was requested, that when possible, one answer per centre was provided to reflect practice within the centre rather than an individual clinician opinion. The survey was distributed in 7 European countries.

## Results

The survey received 95 responses (United Kingdom = 42, Belgium = 18, Netherlands = 14, France = 8, Germany = 7, Switzerland = 5, Poland = 1) from 93 centres and was re-sent to non-responders. The overall response to the survey was 66% (95/143). In some centres it was not possible to provide a consensus, and therefore in two centres more than one dose and fractionation regime was provided.

### Thoracic radiotherapy

Before the publication of CREST only 24 (25%) centres delivered thoracic radiotherapy routinely to patients who responded to chemotherapy, compared to the current practice of 81 (85%). Three clinical scenarios were provided to the surveyed centres with regards to response to initial chemotherapy, see Table 1. The largest increase in the use of thoracic radiotherapy was in patients with asymptomatic residual thoracic disease after chemotherapy.

**Table 1:** Centres delivering thoracic radiotherapy routinely to patients who respond to chemotherapy.

	Pre CREST study (*n* = 24)	Post CREST study (*n* = 81)
Does your centre give thoracic radiotherapy routinely? (*n* = 95)	25% (24/95)	85% (81/95)
If thoracic RT applied, in which scenario is it used?		
• Patients with symptomatic residual disease	92% (22/24)	92% (74/80)
• Patients with asymptomatic central residual disease	79% (19/24)	93% (75/81)
• Patients with no central disease	42% (10/24)	49% (40/81)

An upper limit of performance status (ECOG2) is applied to select patients for thoracic radiotherapy in 54 (79%) centres.

**Fig. 1:**
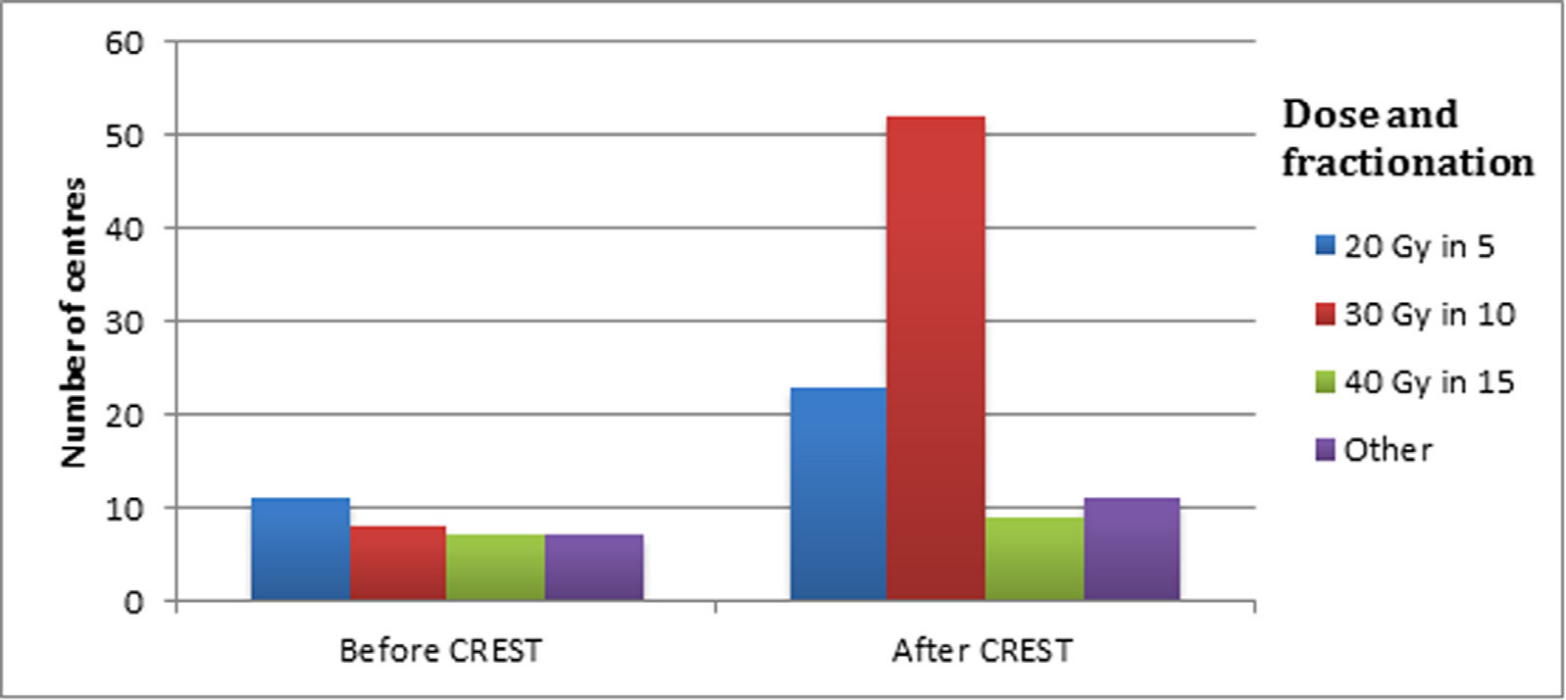
Dose and fractionation used for ES-SCLC patients before and after the publication of the CREST study.

Prior to the publication of the trial, thoracic radiotherapy dose fractionation varied widely, but now the dose delivered in the experimental arm of CREST (30 Gy in 10 fractions) is prescribed in 52 (69%) centres, see Fig. 1.

In the 18 (18%) centres who did not implement thoracic radiotherapy after the publication of the CREST trial, a variety of explanations were given, including the primary endpoint of the study not being met, or the difference in survival not being clinically meaningful, but no single reason stood out.

### Prophylactic cranial irradiation

In patients who have responded to chemotherapy, 92 (97%) centres give prophylactic cranial irradiation routinely. Of these, 45 (49%) deliver 25 Gy in 10 fractions and 40 (43%) deliver 20 Gy in 5 fractions.

An upper age limit was applied in 30 (33%) centres for selection of patients for prophylactic cranial irradiation. Most commonly an upper age limit of 75 was applied in 18 (60%) centres.

An upper limit for performance status was applied in 81 (88%) centres, most commonly ECOG 2.

### Staging investigations

The practice that differed most between centres was which routine staging investigations were performed before chemotherapy. A computed tomography (CT) thorax and abdomen was performed in 61 (64%) centres, CT thorax, abdomen and pelvis in 27 (28%) centres, Positron Emission Tomography (PET) scan in 33 (35%) centres, imaging of the brain (CT or MRI) in 55 (57%) centres and an Isotope bone scan in 13 (14%) centres. Interestingly, in the United Kingdom CT/MRI imaging of the brain was only performed in 8 (14%) centres compared to 51 (86%) of centres outside the United Kingdom.

### Future research

When asked which research question was most important to study next in this group of patients, responses varied widely from adding targeted agents/immunotherapy to thoracic radiotherapy, both increasing the dose of thoracic radiotherapy and using radiotherapy and/or SABR to treat metastatic sites and determining which patient groups would benefit from thoracic radiotherapy.

## Discussion

Our results show an increase in the use of thoracic radiotherapy in patients with ES-SCLC, suggesting the CREST trial has changed practice. The largest increase was in patients with asymptomatic residual thoracic disease after chemotherapy. The dose and fractionation schedule used in the trial has been widely adopted across Europe. The survey also shows high consistency in European practice in the use of prophylactic cranial irradiation. The staging procedures were very heterogeneous with limited use of PET-CT and brain imaging, which can impact on the outcome of these patients after thoracic radiotherapy.

A question raised in correspondence to the Lancet [7], [9] following the publication of CREST and from a number of the responders to our questionnaire was which subgroups of patients will benefit most from thoracic radiotherapy in ES-SCLC. This has been initially addressed by further analysis of patients in the CREST trial. In the trial, patients were stratified by the presence or absence of intrathoracic disease after chemotherapy and further analysis demonstrated a statistically significant overall survival benefit in patients with residual intrathoracic disease who received thoracic radiotherapy (hazard ratio 0.81, 95% CI 0.66–1.00, *p* = 0.044) [8]. Additional data on sites and number of metastases has been collected from 260 patients across the top 9 recruiting centres in the CREST trial (53% of the 495 study patients were included) [10]. The overall survival (*p* = 0.02) and progression free survival (PFS) (*p* = 0.04) were significantly better in patients with 2 or fewer metastases, with significantly worse overall survival if liver (*p* = 0.03) and/or bone metastases (*p* = 0.04) were present. The additional analysis suggests that future studies evaluating more intensive thoracic and extra-thoracic radiotherapy in ES-SCLC should focus on patients with fewer than 3 distant metastases.

## Funding

The CREST trial was funded by the Dutch Cancer Society (CKTO), Dutch Lung Cancer Research Group, Cancer Research UK, Manchester Academic Health Science Centre Trials Coordination Unit, and the UK National Cancer Research Network.

## Conflicts of interest

The authors declare no conflicts of interest.

## Appendix 1 Questionnaire

1. In which country do you practice?
2. Which centre are you from?
3. Did your centre take part in the REST study?
  a) Yes
  b) No
4. In patients with extensive stage small-cell lung cancer (ES-SCLC) which staging investigations are routinely carried out before chemotherapy?
  a) CT thorax & abdomen
  b) CT thorax, abdomen & pelvis
  c) CT brain
  d) MRI brain
  e) Isotope bone scan
  f) PET scan
  g) Other, please specify
5. In patients with ES-SCLC which staging investigations are routinely carried out after chemotherapy?
  a) CT thorax & abdomen CT thorax & abdomen
  b) CT thorax, abdomen & pelvis
  c) CT brain
  d) MRI brain
  e) Isotope bone scan
  f) PET scan
  g) Other, please specify
6. Do you use prophylactic cranial irradiation (PCI) routinely in patients with ES-SCLC who have responded to chemotherapy?
  a) Yes
  b) No
7. What dose and fractionation do you use?
  a) 20 Gy in 5 fractions
  b) 25 Gy in 10 fractions
  c) 30 Gy in 12 fractions
  d) Other, please specify
8. Do you apply an upper age limit for PCI in ES-SCLC?
  a) Yes
  b) No
9. What is your upper age limit for PCI?
  a) 70 years old
  b) 75 years old
  c) 80 years old
  d) Other, please specify
10. Do you restrict the use of PCI in ES-SCLC based on performance status?
  a) Yes
  b) No
11. What is your upper limit for performance status using ECOG?
  a) 0
  b) 1
  c) 2
  d) 3
  e) Other, please specify
12. Before the publication of the (C)REST study did your centre give thoracic radiotherapy routinely to patients who had responded to chemotherapy?
  a) Yes
  b) No
13. Before the publication of the (C)REST study did your centre give thoracic radiotherapy to patients who had responded to chemotherapy with symptomatic residual disease?
  a) Yes
  b) No
14. What dose and fractionation did you use (symptomatic residual disease)?
  a) 20 Gy in 5 fractions
  b) 30 Gy in 10 fractions
  c) 40 Gy in 15 fractions
  d) Other, please specify
15. Before the publication of the (C)REST study did your centre give thoracic radiotherapy to patients who had responded to chemotherapy with asymptomatic central residual disease?
  a) Yes
  b) No
16. What dose and fractionation did you use (asymptomatic central residual disease)?
  a) Yes
  b) No
17. Before the publication of the (C)REST study did your centre give thoracic radiotherapy to asymptomatic patients who had responded to chemotherapy with no central residual disease?
  a) Yes
  b) No
18. What dose and fractionation did you use (asymptomatic no central residual disease)?
  a) 20 Gy in 5 fractions
  b) 30 Gy in 10 fractions
  c) 40 Gy in 15 fractions
  d) Other, please specify
19. Before the publication of the (C)REST study what was your preferred time interval between completion of chemotherapy and starting thoracic radiotherapy?
  a) 3 weeks
  b) 4 weeks
  c) 6 weeks
  d) 8 weeks
  e) Other, please specify
20. Since the publication of the (C)REST study does your centre use thoracic radiotherapy to patients who have responded to chemotherapy?
  a) Yes
  b) No
21. Since the publication of the (C)REST study does your centre give thoracic radiotherapy to patients who have responded to chemotherapy with symptomatic residual disease?
  a) Yes
  b) No
22. What dose and fractionation did you use (symptomatic residual disease)?
  a) 20 Gy in 5 fractions
  b) 30 Gy in 10 fractions
  c) 40 Gy in 15 fractions
  d) Other, please specify
23. Since the publication of the (C)REST study does your centre give thoracic radiotherapy to patients who have responded to chemotherapy with asymptomatic central residual disease?
  a) Yes
  b) No
24. What dose and fractionation did you use (symptomatic residual disease)?
  a) 20 Gy in 5 fractions
  b) 30 Gy in 10 fractions
  c) 40 Gy in 15 fractions
  d) Other, please specify
25. Since the publication of the (C)REST study does your centre give thoracic radiotherapy to asymptomatic patients who have responded to chemotherapy with no central residual disease?
  a) Yes
  b) No
26. What dose and fractionation did you use (asymptomatic no central residual disease)?
  a) 20 Gy in 5 fractions
  b) 30 Gy in 10 fractions
  c) 40 Gy in 15 fractions
  d) Other, please specify
27. In your current practice do you apply an upper age limit when giving thoracic radiotherapy in ES-SCLC?
  a) Yes
  b) No
28. What is your upper age limit?
  a) 70 years old
  b) 75 years old
  c) 80 years old
29. Do you restrict the use of thoracic RT in ES-SCLC based on performance status?
  a) Yes
  b) No
30. What is your upper limit for performance status using ECOG?
  a) 0
  b) 1
  c) 2
  d) 3
  e) Other, please specify
31. After the publication of the (C)REST study what is your preferred time interval between completion of chemotherapy and starting thoracic radiotherapy?
  a) 3 weeks
  b) 4 weeks
  c) 6 weeks
  d) 8 weeks
  e) Other, please specify
32. If thoracic radiotherapy is not going to be implemented in your centre following the publication of the (C)REST study, please tick if any of the explanations apply below or we would be grateful if you could expand using the free text?
  a) Not applicable, my centre has implemented thoracic RT
  b) Difference in survival not clinically meaningful
  c) Primary endpoint of the study not met
  d) Concerns regarding toxicity of thoracic radiotherapy
  e) Confirmatory trial needed before a change in practice is implemented
  f) Other, please specify
33. Following the publication of the (C)REST study which research question do you think is most important in this group of patients?
  a) Increasing the dose of thoracic radiotherapy
  b) Using radiotherapy and/or SABR to treat metastatic sites
  c) Both increasing the dose of thoracic radiotherapy and using radiotherapy and/or SABR to treat metastatic sites
  d) Delivery of thoracic radiotherapy concurrently with chemotherapy
  e) Addition of targeted agents/immunotherapy to thoracic radiotherapy
  f) Other, please specify
34. Please make any general comments on the use of thoracic radiotherapy in patients who have responded to chemotherapy in ES-SCLC, and/or any difficulties in answering the survey.
